# Identification of a Prognostic Hypoxia-Associated Gene Set in IDH-Mutant Glioma

**DOI:** 10.3390/ijms19102903

**Published:** 2018-09-25

**Authors:** Philip Dao Trong, Saskia Rösch, Heimo Mairbäurl, Stefan Pusch, Andreas Unterberg, Christel Herold-Mende, Rolf Warta

**Affiliations:** 1 Department of Neurosurgery, Division of Experimental Neurosurgery, Heidelberg University Hospital, Im Neuenheimer Feld 400, 69120 Heidelberg, Germany; Philip.DaoTrong@med.uni-heidelberg.de (P.D.T.); Saskia.Roesch@med.uni-heidelberg.de (S.R.); Andreas.Unterberg@med.uni-heidelberg.de (A.U.); H.Mende@med.uni-heidelberg.de (C.H.-M.); 2 Medical Clinic VII, Sports Medicine, Heidelberg University Hospital, 69120 Heidelberg, Germany; Heimo.Mairbaeurl@med.uni-heidelberg.de; 3 Department of Neuropathology, Institute of Pathology, Heidelberg University Hospital, 69120 Heidelberg, Germany; Stefan.Pusch@med.uni-heidelberg.de; 4 German Consortium of Translational Cancer Research (DKTK), 69120 Heidelberg, Germany

**Keywords:** lower grade glioma, glioma stem cells, isocitrate dehydrogenase mutation, hypoxia gene signature, The Cancer Genome Atlas

## Abstract

Glioma growth is often accompanied by a hypoxic microenvironment favorable for the induction and maintenance of the glioma stem cell (GSC) phenotype. Due to the paucity of cell models of Isocitrate Dehydrogenase 1 mutant (IDH1^mut^) GSCs, biology under hypoxic conditions has not been sufficiently studied as compared to IDH1 wildtype (IDH1^wt^) GSCs. We therefore grew well-characterized IDH1^mut^ (*n* = 4) and IDH1^wt^ (*n* = 4) GSC lines under normoxic (20%) and hypoxic (1.5%) culture conditions and harvested mRNA after 72 h. Transcriptome analyses were performed and hypoxia regulated genes were further analyzed using the expression and clinical data of the lower grade glioma cohort of The Cancer Genome Atlas (LGG TCGA) in a confirmatory approach and to test for possible survival associations. Results show that global expression changes were more pronounced in IDH1^wt^ than in IDH1^mut^ GSCs. However, when focusing on known hypoxia-regulated gene sets, enrichment analyses showed a comparable regulation in both IDH1^mut^ and IDH1^wt^ GSCs. Of 272 significantly up-regulated genes under hypoxic conditions in IDH1^mut^ GSCs a hypoxia-related survival score (HRS-score) of five genes (*LYVE1*, *FAM162A*, *WNT6*, *OTP*, *PLOD1*) was identified by the Least Absolute Shrinkage and Selection Operator (LASSO) algorithm which was able to predict survival independent of age, 1p19q co-deletion status and WHO grade (II vs. III) in the LGG TCGA cohort and in the Rembrandt dataset. Altogether, we were able to identify and validate a novel hypoxia-related survival score in IDH1^mut^ GSCs consisting of five hypoxia-regulated genes which was significantly associated with patient survival independent of known prognostic confounders.

## 1. Introduction

The discrimination of glioma into IDH1^wt^ and IDH1^mut^ tumors has marked an important milestone due to its profound clinical impact [1]. Whereas patients developing glioblastoma (GBM), the most common and malignant glioma in adults, have a dismal prognosis and usually die after about 15 months with the current standard of care (maximal safe surgical resection, followed by radiotherapy plus concomitant and adjuvant chemotherapy with temozolomide), lower grade glioma (LGG) patients show a much better clinical course [2,3,4,5]. To understand the differences in underlying tumor biology the international consortium ‘The Cancer Genome Atlas’ (TCGA) used genome-wide data from multiple platforms to identify molecular subtypes for GBM and LGG with prognostic relevance [6,7]. The main distinction is the IDH mutation which has a very high incidence within LGG patients of up to 80% and is virtually absent in primary GBM [7]. This gain of function mutation leads to the production of the onco-metabolite 2-hydroxyglutarate (2HG) which interferes with many pathways and its opposing appearance suggests that IDH^mut^ and IDH1^wt^ glioma are in fact biologically different tumor types [8,9].

As a consequence, the traditional WHO classification has been revisited in 2016 and has implemented molecular markers in addition to histology [10]. Among IDH^mut^ gliomas, the WHO classification system distinguishes three grades (°II-IV) based on histopathologic features. Within each grade, IDH^mut^ gliomas have a significantly better prognosis compared with their IDH^wt^ counterparts. Although the underlying mechanisms have not been fully elucidated the discovery of the *IDH* mutation had a fundamental impact on glioma research [7,11,12,13].

There is a large body of evidence that a subpopulation of tumor cells which are reminiscent of normal neural stem cells and therefore called glioma stem cells (GSCs) are driving tumor formation and resistance to chemoradiation [14,15,16,17]. Moreover, their microenvironment seems to profoundly contribute to the observed treatment resistance which especially applies to the effect of low oxygen tension [18]. Hypoxia has been shown to promote and stabilize stem cell-like properties, such as invasiveness, differentiation, proliferation, self-renewal capacity, and resistance to radiochemotherapy and is found in solid tumors where growth outruns vessel supply and oxygenation [19,20,21,22,23,24,25]. This translates into a significant survival disadvantage in cancers of the uterine cervix, head and neck, and soft tissue sarcomas [26,27,28,29,30]. In lower and high grade-glioma stratification for *IDH1* status showed conflicting results. While some investigators report a transcriptional activation of hypoxia-related genes, others describe the opposite effect most likely due to different methodological approaches [31,32,33,34,35]. Since IDH1 is part of the tricarboxylic acid (TCA) cycle and plays a major role in energy and oxygen metabolism, it is crucial to understand how hypoxia alters the phenotype of IDH^mut^ glioma cells as compared to its wildtype counterpart [9].

In the present study we cultivated GSCs derived from human IDH1^mut^ and IDH^wt^ tumor tissues instead of cells where IDH mutations were artificially introduced and thus did not represent the natural genetic background. This allowed us to gain relevant insights on a whole transcriptomic level and to identify a gene set of five hypoxia-regulated genes in IDH^mut^ GSCs prognosticating patient survival independent of known confounders such as age, codel-subtype or WHO grade.

## 2. Results

### 2.1. Distinct Response to Hypoxia of IDH1^mut^ and IDH1^wt^ Glioma Stem Cells

To analyze the effect of hypoxia on the transcriptome of IDH1^mut^ glioma in comparison to IDH1^wt^ GBM we made use of human IDH1^wt^ (*n* = 4) as well as of IDH1^mut^ (*n* = 4) GSC lines (Supplementary Materials Figure S1, Table 1). All four IDH1^wt^ GSCs were derived from primary GBM. As for IDH1^mut^ GSCs, one cell line was established from an anaplastic oligodendroglioma and three from secondary GBM. A maintained 2HG secretion in the supernatant of all IDH1^mut^ GSCs as a result of the IDH1 mutation could be confirmed by an enzymatic assay with concentrations ranging from 9.4–1792.1 µM, whereas in IDH1^wt^ GSCs 2HG levels were below the detection limit (Table 1) [36]. To induce hypoxic conditions, we cultivated the cells for 72 h under oxygen deprivation (1.5% O_2_) while cultivating control cells under standard normoxic conditions (~20% O_2_).

**Figure 1:**
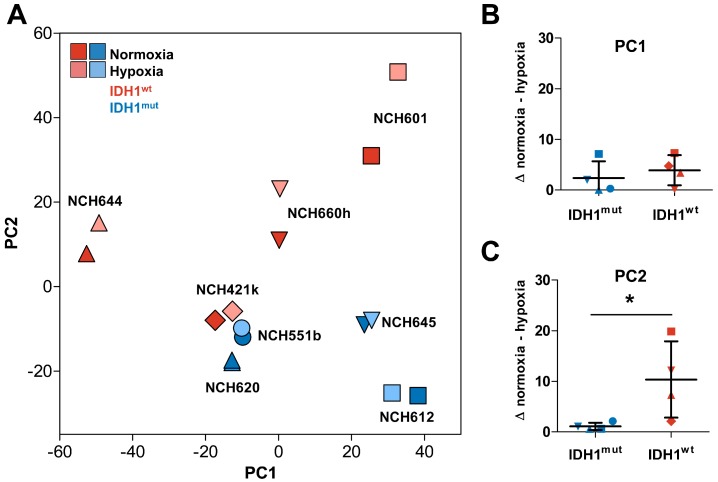
Transcriptome analysis of IDH1^mut^ and IDH1^wt^ glioma stem cells (GSCs) cultured in normoxia and hypoxia. (**A**) Principal component analysis of global expression changes of IDH1^mut^ (blue symbols) and IDH1^wt^ (red symbols) GSCs. Different geometric forms (triangles, squares, diamonds, circles) represent different cell lines. Hypoxia: light, normoxia: dark colors. (**B**) Absolute difference between hypoxic and normoxic samples in PC1 and (**C**) significant larger difference in PC2 in IDH1^wt^ cells compared to IDH1^mut^ GSCs (* *p* < 0.028).

**Figure 2:**
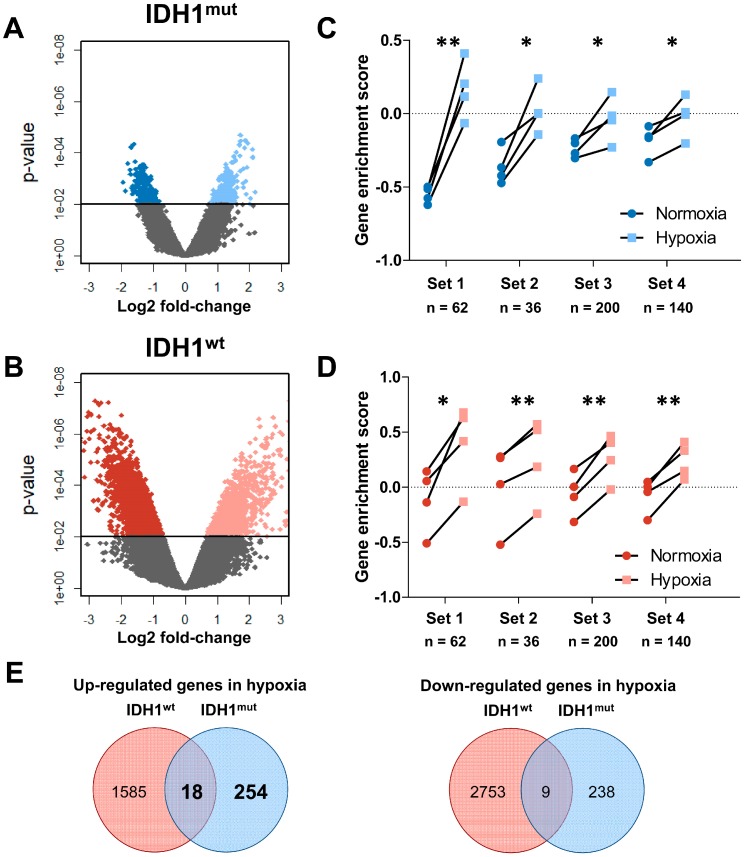
(**A**,**B**) Volcano plots of differentially up- and downregulated genes in IDH1^mut^ (blue) and IDH1^wt^ (red) GSCs. (**C**,**D**) Gene set enrichment analysis (GSE) of known hypoxia signatures as published by Fardin et al. (Set 1) [37], Semenza et al. (Set 2) [39], Liberzon et al. (Set 3) [38], and Qi et al. (Set 4) [40] for IDH1^mut^ (blue) and IDH1^wt^ (red) GSCs. A higher GSE score indicates a better correlation with the published gene set and considered significant if *p* < 0.05 (paired Student’s *t*-Test, * *p* < 0.05; ** *p* < 0.01) (n = number of genes in set). (**E**) Venn diagrams showing the overlap of differentially expressed genes in hypoxia of IDH^mut^ and IDH1^wt^ GSCs.

To analyze the transcriptome changes in response to hypoxia we conducted mRNA microarrays followed by a principal component analysis (PCA, Figure 1A). Interestingly, the hypoxic as well as the normoxic samples of the same GSC line showed only small differences in the principal component (PC) 1, which represents the highest variance in the data, regardless of their IDH1 status. This suggests that the individual genetic background of each glioma cell line causes larger transcriptomic changes than the IDH1 status or the oxygen levels (Figure 1B). Nevertheless, PC2 clearly distinguishes IDH1^mut^ (upper area) and IDH1^wt^ cells (lower area). However, when analyzing the difference in the PC2 we found a uniform increase of PC2 in hypoxia in IDH1^wt^ cells, whereas IDH1^mut^ cells showed only a small shift which was significantly lower as compared to IDH1^wt^ GSCs (Figure 1C, *p* = 0.028). This suggests that the global transcriptome of IDH1^mut^ GSCs seems to react to oxygen levels to a much lesser extent than in IDH1^wt^ GSCs. This hypothesis was sustained when we analyzed the differentially regulated genes in response to hypoxia in IDH1^mut^ and IDH1^wt^ GSCs (Figure 2A,B). Here, we found 519 hypoxia-regulated genes in IDH1^mut^ GSCs (*p* = 0.01, *n* = 272 up, *n* = 247 down, Figure 2E) while in IDH1^wt^ GSCs as much as 4365 genes were regulated under hypoxic conditions (*n* = 1603 up, *n* = 2762 down, Figure 2E).

Next we used gene set enrichment analyses (GSEA) to test if we can reproduce the low hypoxic response of IDH1^mut^ GSCs by analyzing published hypoxia gene signatures. To this end we assessed the concerted change of functionally related genes forming a set by the Gene Set Variation Analysis (GSVA) algorithm which computes enrichment scores for every sample (Table 2, Figure 2C,D). Interestingly, this analysis revealed in IDH1^wt^ and IDH1^mut^ GSCs, in contrast to the PCA and the numbers of differentially expressed genes, a comparable response to hypoxia in all four previously published hypoxia associated gene sets [37,38,39,40]. This finding suggests that even though response to hypoxia of IDH1^mut^ GSCs is indeed less pronounced on the transcriptome level those genes that have been observed to be regulated are specific for hypoxic conditions. However, the intersection study of the regulated genes revealed only a small overlap of differentially expressed genes between IDH1^mut^ and IDH1^wt^ GSCs (Figure 2E). Only 18/1857 (1.0%) were commonly up- and 9/3000 (0.3%) commonly downregulated. These results again highlight the fundamental difference in biology of IDH1^mut^ and IDH1^wt^ gliomas.

### 2.2. Hypoxia Score Prognosticates Survival in LGG Patients Independent of 1p19q Co-Deletion Status, WHO Grade and Age

**Figure 3:**
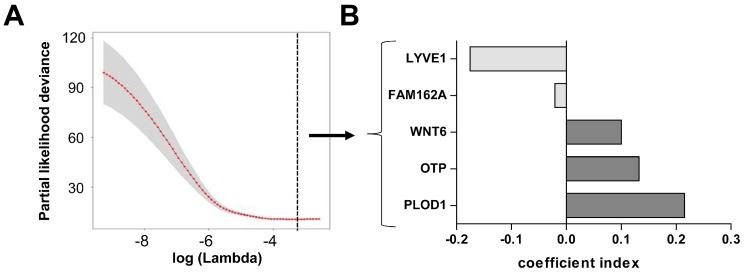
Development of a five gene hypoxia score in the lower grade glioma cohort of The Cancer Genome Atlas (LGG TCGA) dataset. (**A**) The cross-validation error curve shows the regularization path of the Least Absolute Shrinkage and Selection Operator (LASSO) algorithm. The elimination of non-informative genes is visualized by the decreasing prediction error to a minimum (black dotted line) with only the five most important genes left in the Cox ph model (grey shaded area = 5–95% confidence interval). (**B**) These five genes have negative and positive coefficients, implicating that namely *LYVE1* and *FAM162A* correlate with a better survival when up-regulated, whereas *WNT6*, *OTP*, and *PLOD1* correlate with a worse survival.

Since hypoxia has often been related to the outcome of patients we sought to develop a hypoxia-related gene signature in LGG patients. Because this has already been extensively studied in IDH1^wt^ GSCs we solely focused on the 272 genes up-regulated in hypoxia in IDH1^mut^ GSCs (Supplementary Materials Table S1). To this end, we used the machine learning technique LASSO which at the same time selects unsupervised from the 272 input genes the most informative ones and fits a Cox ph regression model with an optimal prediction power for all 395 IDH1^mut^ patients in the LGG TCGA dataset, regardless of their 1p19q co-deletion status and WHO grade (II and III). The process of elimination of non-informative genes is visualized by the cross-validation error curve in Figure 3A, where all genes are included on the left side and the prediction error drops to a minimum (black dotted line) with only the five most important genes left in the model (Figure 3A).

Interestingly, LASSO selected genes with negative and positive coefficients implicating that namely *LYVE1* and *FAM162A* are associated with a better survival when up-regulated, whereas *WNT6*, *OTP*, and *PLOD1* are associated with a worse survival (Figure 3B). We could not find classical hypoxia response elements in the promoter sequences of these genes, but CHIPseq analysis to identify HIF2a binding sites revealed an association to *OTP* [41]. Noteworthy, *PLOD1*, mediating crosslinking of collagen fibers, is the only commonly up-regulated gene in both IDH^mut^ and IDH1^wt^ GSCs under hypoxia and has shown to be regulated by HIF1alpha in breast cancer cells leading to increased invasion and metastasis [42]. The survival associations could be validated on a single gene level by log-rank tests after classifying the patients into high or low mRNA expression according to the median (Supplementary Materials Figure S2). Based on these selected genes we created a hypoxia-related survival score (HRS-score), which incorporates the expression changes of all five candidate genes to prognosticate the survival for a given patient.

**Figure 4:**
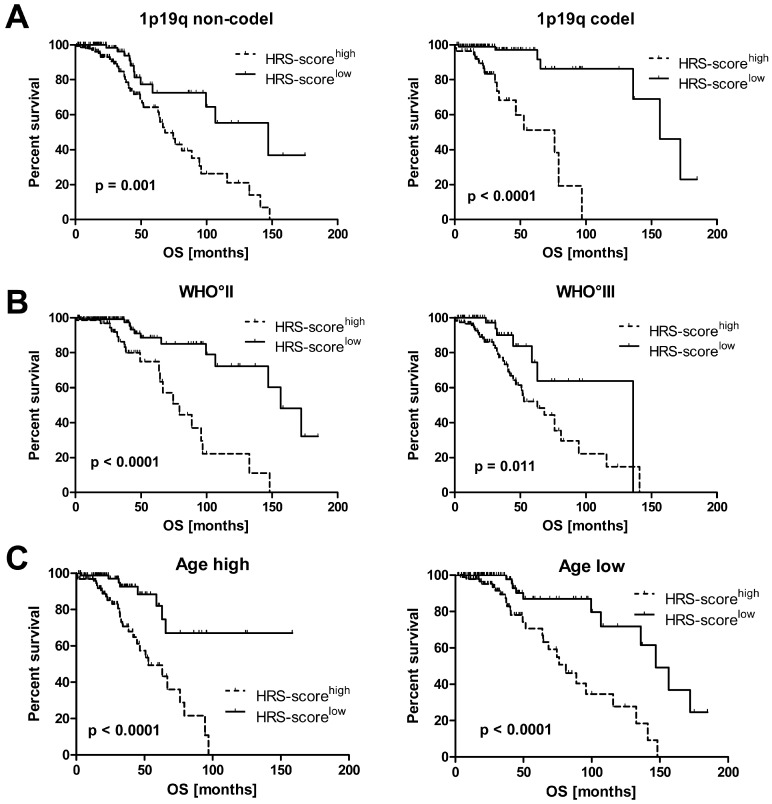
Survival analysis of the LGG TCGA cohort when median splitting the following subgroups into HRS-score^high^ and HRS-score^low^: (**A**) 1p19q co-deletion status, (**B**) WHO grade II and III, (**C**) Age high and low (median splitted) (ticks = censored events, OS: overall survival).

Next, we confirmed in univariate Kaplan-Meier analyses that the HRS-score predicts the survival of IDH^mut^ LGG patients independent of molecular and clinical covariates such as 1p19q co-deletion status, WHO grade (II and III) and patient age at diagnosis for the used LGG TCGA cohort (Figure 4).

**Figure 5:**
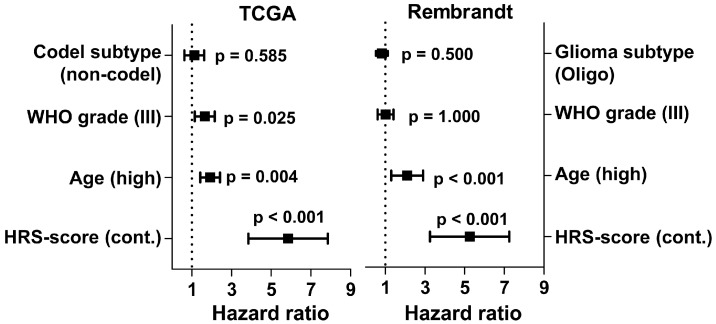
Clinical, histological and molecular confounders were analyzed together with the continuous HRS-score in a multivariate Cox ph model in the TCGA and Rembrandt dataset. A high HRS-score proved to be a highly significant predictor of shorter patient survival independent of age, histology, codel status, and WHO grade in both datasets.

When including age, codel subtype and WHO grade in a multivariate Cox ph analysis we found that a high HRS-score is a highly significant predictor of shorter patient survival independent of age, codel status and WHO grade (Figure 5; HR 5.85 [95% CI 2.89, 11.83] vs. 1.92 [1.16, 3.17], 1.12 [0.65, 1.93], and 1.65 [0.96, 2,82], respectively). To validate the HRS-score in an independent dataset we applied it to 120 IDH^mut^ LGG patients from the Rembrandt database. Here, we could again confirm the prognostic value of the HRS-score (Figure 5; HR 5.25 (95% CI 0.64, 1.59) vs. 2.1 (1.36, 3.25), 0.83 (0.49, 1.40), and 1.01 (0.64, 1.59), respectively).

Taken together, the analysis of differentially expressed genes under hypoxia in IDH^mut^ GSCs yielded five genes, whose incorporation into the HRS-score could predict patient survival in the LGG TCGA and Rembrandt cohort independent of prognostic confounders.

## 3. Discussion

The discovery of mutations in *IDH* genes in glioma as compared to their *IDH* wildtype counterpart has marked a fundamental milestone in the understanding of glioma biology [1]. While different oxygen tensions have been extensively studied in IDH1^wt^ glioma cells, due to the wide availability of cell culture models, there is a paucity of IDH1^mut^ glioma models limiting the analysis of hypoxic conditions profoundly. Most experimental models have synthetically introduced the IDH1 mutation into commonly used IDH^wt^ cell lines such as U87MG or U251. A subsequent increase in *HIF1alpha* expression suggests a possible oncogenic mechanism of the *IDH1* mutation by mimicking the tumorigenic effect of the hypoxic microenvironment [31,32,33,43]. In contrast, other groups have demonstrated that HIF1alpha or HIF1alpha-responsive genes are either not affected by an *IDH* mutation or even showed a decrease in the mRNA or protein expression [34,35,44,45,46]. Regarding these heterogeneous results, we sought to add further knowledge by culturing human IDH1^mut^ and IDH1^wt^ GSCs in hypoxia and analyze their transcriptome in their natural genomic background.

Comparative differential gene expression analysis between IDH1^wt^ and IDH1^mut^ GSCs suggested that hypoxia has a bigger impact on IDH1^wt^ GSC on the whole transcriptome than on IDH1^mut^ GSCs. However, after having performed a gene set enrichment analysis of several well-established hypoxia gene signatures, a significant and specific enrichment has been found in IDH1^mut^ GSCs similar to IDH1^wt^ GSCs. This finding suggests that IDH1^mut^ GSCs seem to react to changes in oxygen level in a more specific way than IDH1^wt^ GSCs do. Interestingly, when looking at the list of differentially regulated genes of IDH1^mut^ and IDH1^wt^ GSCs, only 18/1857 (1.0%) were commonly up- and 9/3000 (0.3%) commonly downregulated. The fact that there is only a small fraction of overlapping regulated genes shared by IDH1^mut^ and IDH1^wt^ GSCs further corroborates fundamental differences in their biology.

To clarify the clinical relevance of hypoxia in IDH^mut^ glioma, we further assessed whether the differentially up-regulated genes in GSCs in hypoxia translate into useful clinical parameters using the LGG TCGA dataset. Using the LASSO algorithm, we were able to create a hypoxia-related survival score incorporating the regulation of five genes, whose upregulation (*WNT6*, *OTP*, and *PLOD1*) and downregulation (*LYVE1* and *FAM162A*) show a worse survival in all molecular and histological subgroups (1p19q co-deletion status and WHO II/III, Supplementary Materials Figure S2). Among these, *FAM162A* and *PLOD1* have been listed in the aforementioned gene sets by Fardin et al. (Set 1) and additionally in the gene sets of Liberzon et al. (Set 3) and Qi et al. (Set 4) respectively. *FAM162A* has further been shown to be HIF1alpha responsive and a mediator of the mitochondrial apoptotic pathway [47]. Its elevated expression has been observed in gastric and uterine carcinoma [48,49,50]. *PLOD1*, the only commonly up-regulated gene in IDH1^mut^ and IDH1^wt^ GSCs under hypoxia, mediates crosslinking of collagen fibers and has shown to be regulated by HIF1alpha in breast cancer cells leading to increased invasion and metastasis [42]. The combination of *WNT6* expression with the expression of hypoxia pathway proteins identified a subgroup of hepatocellular carcinoma patients predictive of poor survival [51]. *LYVE1* is known to be an endothelial receptor crucial for dendritic cells to enter lymph vessels by hyaluronan-mediated docking. Targeted deletion of *LYVE1* resulted in a decrease of the capability of dendritic cells to prime CD8+ T cell responses in skin-draining lymph nodes [52]. *OTP* has shown to play an important role in the development of the neurosecretory system in the hypothalamus and in terminal differentiation of neuroblasts [53]. Only recently, *OTP* has been found to be a highly specific marker for pulmonary carcinoid tumors [54,55,56]. As for both *LYVE1* and *OTP*, to our knowledge this study presents the first evidence of hypoxia-related regulation and clinical correlation in IDH1^mut^ glioma patients.

Most importantly in the multivariate analysis the continuous HRS-score proved to be more robust in prognosticating survival than WHO grade or 1p19q co-deletion subtype in the LGG TCGA cohort as well as in the Rembrandt validation cohort. Despite these encouraging results, a limitation of the present study might be that the transfer of our in vitro results to expression data of the LGG TCGA cohort does not represent the oxygenation status of the tumors in vivo. But since we established our cell culture models unbiased from synthetic genetic alterations, we believe that this study was able to reflect hypoxic areas of the tumor as close as an in vitro study can offer and therefore might be of value in better understanding IDH1^mut^ glioma biology in the context of hypoxia. However, functional experiments investigating selected genes for their causality to tumorigenesis or the IDH mutation would be highly interesting and should be addressed in further studies. Taken together, this study presents five hypoxia-associated genes whose expression can be incorporated in a novel HRS-score with profound prognostic implications for the survival of IDH^mut^ glioma patients. We hope that the HRS-score could be used to further stratify IDH1^mut^ glioma patients into high and low risk patients in order to better guide treatment timing and estimate their aggressiveness.

## 4. Materials and Methods

### 4.1. Glioma Stem Cell Culture

Four IDH1^wt^ and four IDH1^mut^ patient-derived GSC lines were cultivated as described (Table 1) [54]. In brief, cells were grown as floating neurospheres in DMEM/F-12 medium containing 20% BIT serum-free supplement, basic fibroblast growth factor (bFGF) and epidermal growth factor (EGF) at 20 ng/mL each (all Provitro, Berlin, Germany). To study expression changes, IDH1^mut^ and IDH1^wt^ GSCs were grown under normoxic (~20% O_2_) and hypoxic conditions (1.5% O_2_) as previously reported [24,57]. Hypoxic conditions (1.5% O_2_, 5% CO_2_, 93.5% N_2_) were established in an O_2_- and CO_2_-controlled tissue culture incubator (Nunc, Langenselbold, Germany) as previously described [58]. mRNA was harvested after 72 h using the Qiagen Allprep RNA isolation kit according to the manufacturer’s instructions.

The expression of several stem cell markers including CD133, SOX2, CD44, CSPG4, CD90, and nestin in the IDH1^mut^ GSCs has been shown by Kohanbash et al. [59]. Also for IDH1^wt^ GSCs elevated clonogeneity as well as expression of certain stem cell markers has been shown in previous publications from our laboratory [19,60].

### 4.2. (D)-2-Hydroxyglutarate Measurements

(D)-2-hydroxyglutarate levels were measured in the cell culture supernatant of IDH1^mut^ GSCs as decribed by Balss et al. [36]. In brief (d)-2-hydroxyglutarate dehydrogenase (HGDH) catalyzes the reduction of NAD+ to NADH by oxidation of (d)-2-hydroxyglutarate to α-ketoglutarate. NADH is then detected by a diaphorase/resazurin system which in turn can be measured by the fluorescent product resorufin which is exited at 540 nm and detected at 610 nm.

### 4.3. Microarray Analysis and Data Normalization

1 μg of total RNA from normoxic and hypoxic cells was submitted to the Genomics Core Facilities of the German Cancer Research Center (DKFZ, Heidelberg, Germany) for microarray analysis. After purification, reverse transcription into cDNA and labeling according to the Illumina protocol, samples were hybridized to Human HT-12 V.4.0 arrays (Illumina, San Diego, CA, USA). Raw data can be accessed in the NCBI GEO repository under the accession number GSE118683. Raw intensity data were obtained after image analysis of the fluorescent spot intensity reads. All preprocessing and normalization steps were performed in the “R” programming environment (available online: www.r-project.org). Inter-array normalization was conducted using vsn normalization in the vsn package. Differential gene expression was assessed by a paired test with the limma package. Survival analysis was conducted within the survival package. Gene set enrichment analysis for hypoxia related gene sets in the C2 (curated gene sets) and C5 (GO gene sets) collections of the MSigDB library was conducted by the GSVA package.

### 4.4. Lower Grade Glioma TCGA and Rembrandt Datasets

RNAseq data from the TCGA Lower Grade Glioma cohort were downloaded from firehose (available online: https://gdac.broadinstitute.org). Raw data were voom normalized by the help of the limma package. Of 515 patients, only IDH^mut^ cases were considered for further clinical analysis (*n* = 395). In line with The Cancer Genome Atlas Research Network, we analyzed survival in the following IDH mutated subgroups: LGG with 1p/19q co-deletion (*n* = 159), LGG without 1p19q co-deletion (*n* = 236) and LGG WHO° II (*n* = 214) and WHO° III (*n* = 181) (Supplementary Materials Figure S3). The microarray raw data of Rembrandt were downloaded from the ArrayExpress archive (accession number: E-MTAB-3073). CEL files were processed with the affy package and vsn normalized in R. Clinical annotation data were retrieved from the G-DOC database (available online: https://gdoc.georgetown.edu/gdoc/). Based on the clinical data we selected patients with either astrocytoma or oligodendroglioma and completed survival data.

### 4.5. Statistics

LASSO (least absolute shrinkage and selection operator) is a linear regression which performs both, variable selection and regularization in a given dataset to fit a model that describes a linear correlation of a response variable (here survival) and several explanatory variables (here genes) [61]. This machine learning algorithm is very powerful and fits better models than conventional Cox ph analyses if there are many explanatory variables and a relatively low amount of samples. Nowadays this represents a typical problem for datasets generated by high throughput techniques [62]. The assumption of LASSO is that there is a reduced subset of explanatory variables within the population of all explanatory variables which is highly informative to model the dependent variable. To select this subset LASSO starts a process in which it iterates over a range of penalty factors (lambda) which is applied to the coefficients of the explanatory variables. This forces some of the coefficients to become zero which removes them effectively from the linear equation leading to the result of such a model [61]. To find the best value of lambda, LASSO is used in combination with the widely used cross-validation, which divides a population into training and test samples. The training samples are used to fit a model whose prediction accuracy is then tested on the test samples. The lambda with the lowest prediction error represents the best model and remaining explanatory variables and their respective coefficients can be extracted [61].

Furthermore, LASSO was adopted to fit Cox ph survival models and is implemented in the broadly used glmnet package within the statistical software environment R [62]. Gene selection for survival prediction of LGG TCGA patients was done by the cv.glmnet function within the glmnet package (family = cox, alpha = 1). 20-fold cross-validation was used to assess the prediction error for the regularization path. As recommended in the glmnet vignette we used the minimum of the prediction error curve to select lambda and extracted the corresponding genes and their coefficients [63]. Expression data were z-normalized prior to LASSO regression using the scale function. Hypoxia-related survival scores (HRS-score) of TCGA and Rembrandt samples were predicted using the predict.cv.glmnet function of the glmnet package based on the previously fitted model as follows:

HRS-score = Z_LYVE1_ × − 0.175 + Z_FAM162A_ × − 0.021 + Z_WNT6_ × 0.100 + Z_OTP_ × 0.132 + Z_PLOD1_ × 0.132 (1)

“Z” depicts the z-normalized raw expression values obtained by subtracting the population mean from an individual expression value and then dividing the difference by the population standard deviation.

Statistical analyses in boxplots were performed using the “Prism 5” software (GraphPad, La Jolla, California). *p* values for differences in survival between the groups were calculated with the log-rank (Mantel Cox) test, whereas the median of expression was used to dichotomize the cohort and to define “high” vs. “low”. Univariate and multivariate Cox regression analyses were performed to determine the prognostic significance of selected candidate genes and the hypoxia score. A *p*-value ≤ 0.05 was considered significant. Student’s *t*-Test was performed for the gene set enrichment analysis between GSCs cultured in normoxia and hypoxia for IDH1^mut^ and IDH1^wt^.

## Figures and Tables

**Table 1:** Description of glioma stem cell lines.

GSC Line	Histology	IDH1 Status	2HG (µM)	Gender	Age
NCH551b	sGBM	mut	139.6	m	48
NCH612	Oligo III	mut	40.2	m	47
NCH620	sGBM	mut	9.4	f	35
NCH645	sGBM	mut	1792.1	m	66
NCH421k	pGBM	wt	b.d.l.	m	77
NCH601	pGBM	wt	b.d.l.	m	84
NCH644	pGBM	wt	b.d.l.	f	74
NCH660h	pGBM	wt	b.d.l.	f	81

p/sGBM = primary/secondary glioblastoma, Oligo = Oligodendroglioma, mut = mutant, wt = wildtype, m = male, f = female, b.d.l. = below detection limit.

**Table 2:** Gene set enrichment analysis (GSEA) by known hypoxia sets.

	Gene Enrichment Set	Fardin (n = 62)	Semenza (n = 36)	Liberzon (n = 200)	Qi (n = 140)
**IDH1^mut^**	mean normoxia	−0.553	−0.364	−0.236	−0.185
	mean hypoxia	0.166	0.024	−0.036	−0.018
	difference in mean	−0.719	−0.389	−0.200	−0.167
	*p*-value	**0.003**	**0.021**	**0.048**	**0.031**
**IDH1^wt^**	mean normoxia	−0.112	0.012	−0.060	−0.073
	mean hypoxia	0.398	0.258	0.273	0.239
	difference in mean	−0.510	−0.246	−0.333	−0.313
	*p*-value	**0.017**	**0.005**	**0.006**	**0.008**

*n* = number of genes in signature.

## Supplementary Materials

Supplementary materials can be found at http://www.mdpi.com/1422-0067/19/10/2903/s1.

## Abbreviations

2HG	2-hydroxyglutarate
GBM	Glioblastoma
Cox ph	Cox proportional hazard
GSC	Gliom stem cells
GSEA	Gene set enrichment analysis
HR	Hazard ratio
HRS-score	Hypoxia-related survival score
IDH(1)^mut^	Isocitrate Dehydrogenase (1) mutated
IDH(1)^wt^	Isocitrate Dehydrogenase (1) wildtype
LASSO	Least Absolute Shrinkage and Selection Operator
LGG	Lower grade glioma
OS	Overall Survival
PC1/2	Principal Component 1/2
PCA	Principal Component Analysis
Rembrandt	REpository for Molecular BRAin Neoplasia DaTa
TCGA	The Cancer Genome Atlas
