# Supplementary figures and images for: Identification of a Prognostic Hypoxia-Associated Gene Set in IDH-Mutant Glioma
Source: Int J Mol Sci. 2018 Sep 25;19(10):2903. doi: 10.3390/ijms19102903 (PMC6212863; doi:10.3390/ijms19102903)

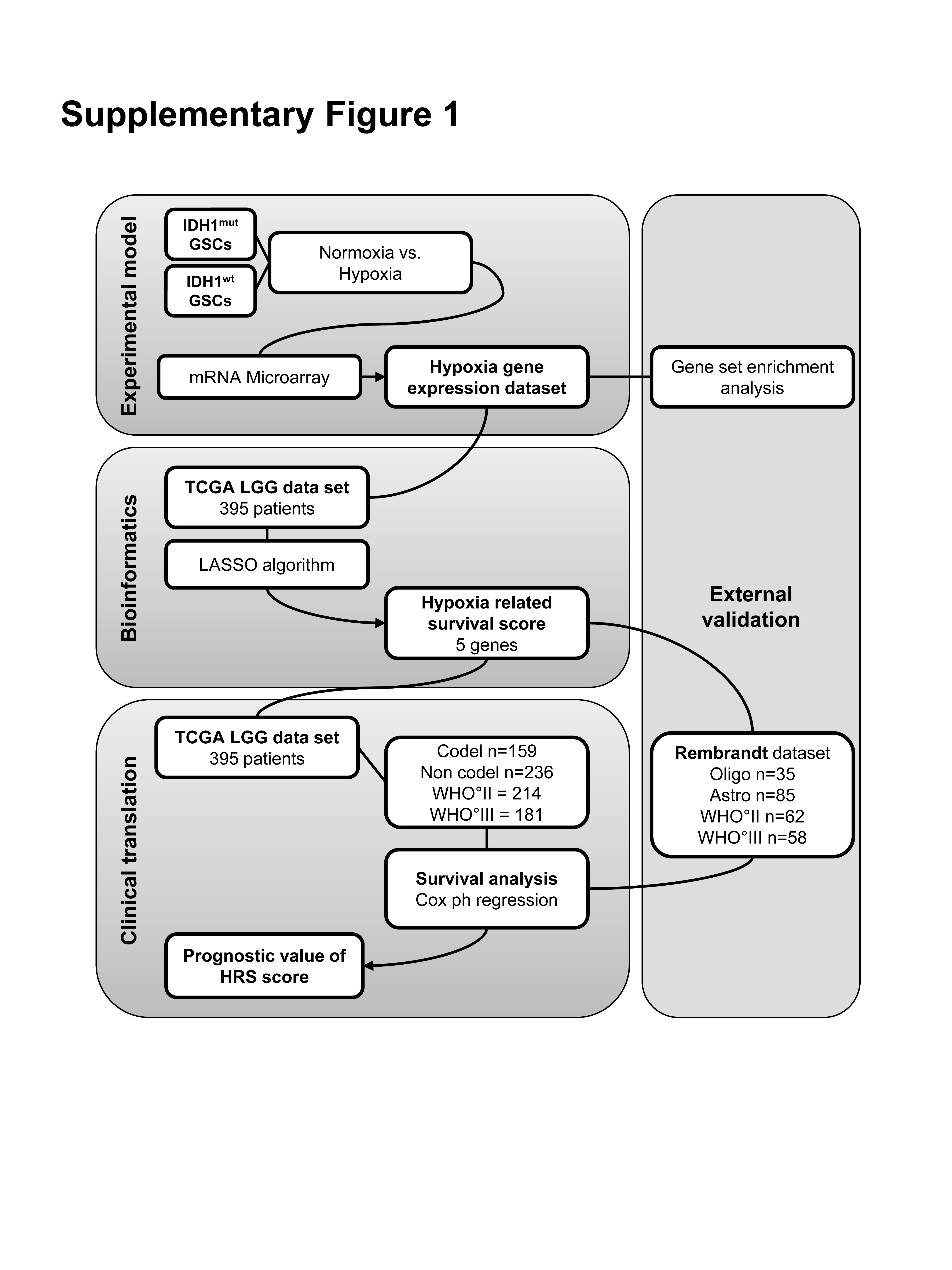

Supplement: Supplementary file 1 [file ijms-19-02903-s001.zip › SupplFigure1.jpg]

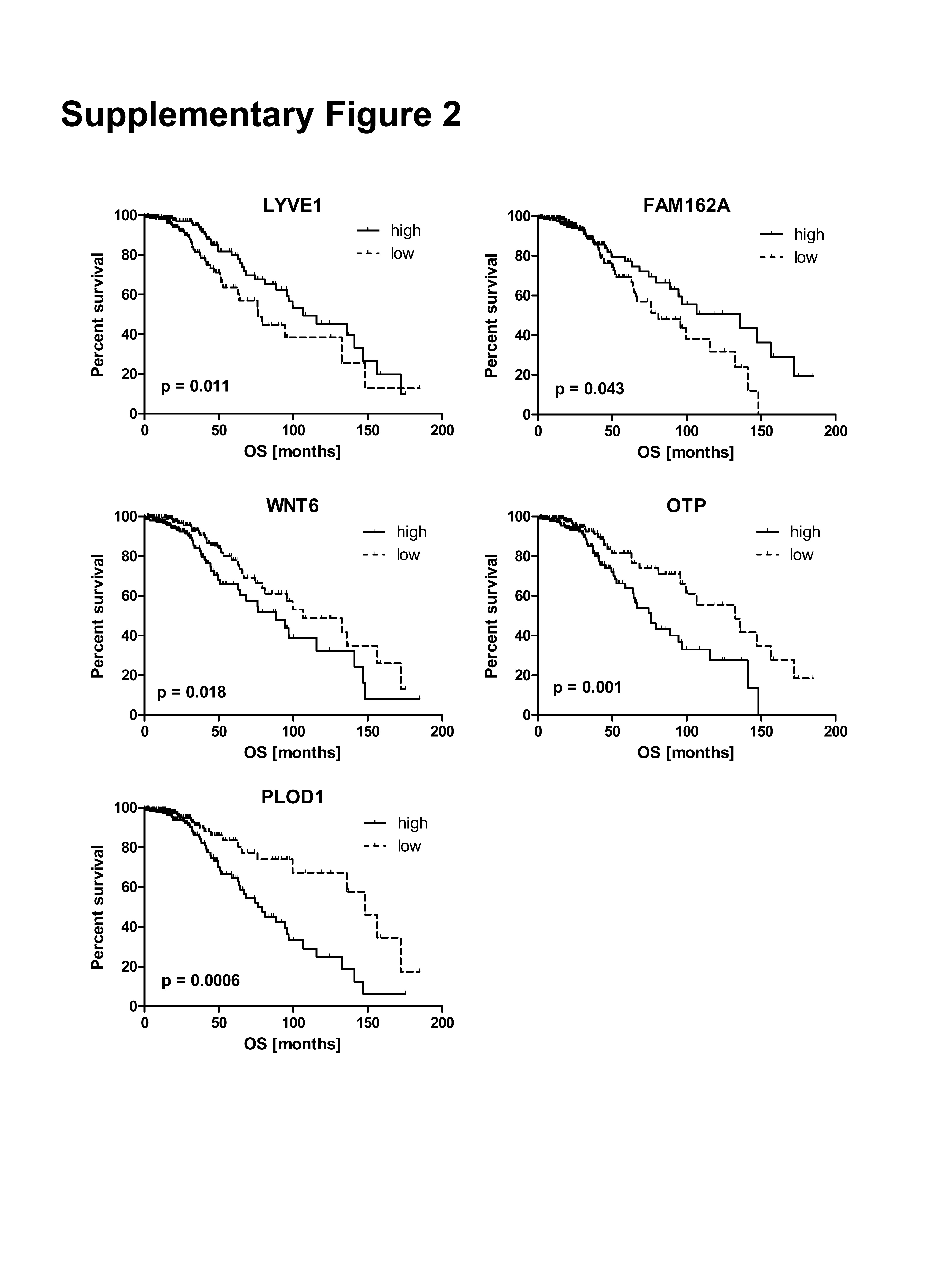

Supplement: Supplementary file 1 [file ijms-19-02903-s001.zip › SupplFigure2.jpg]

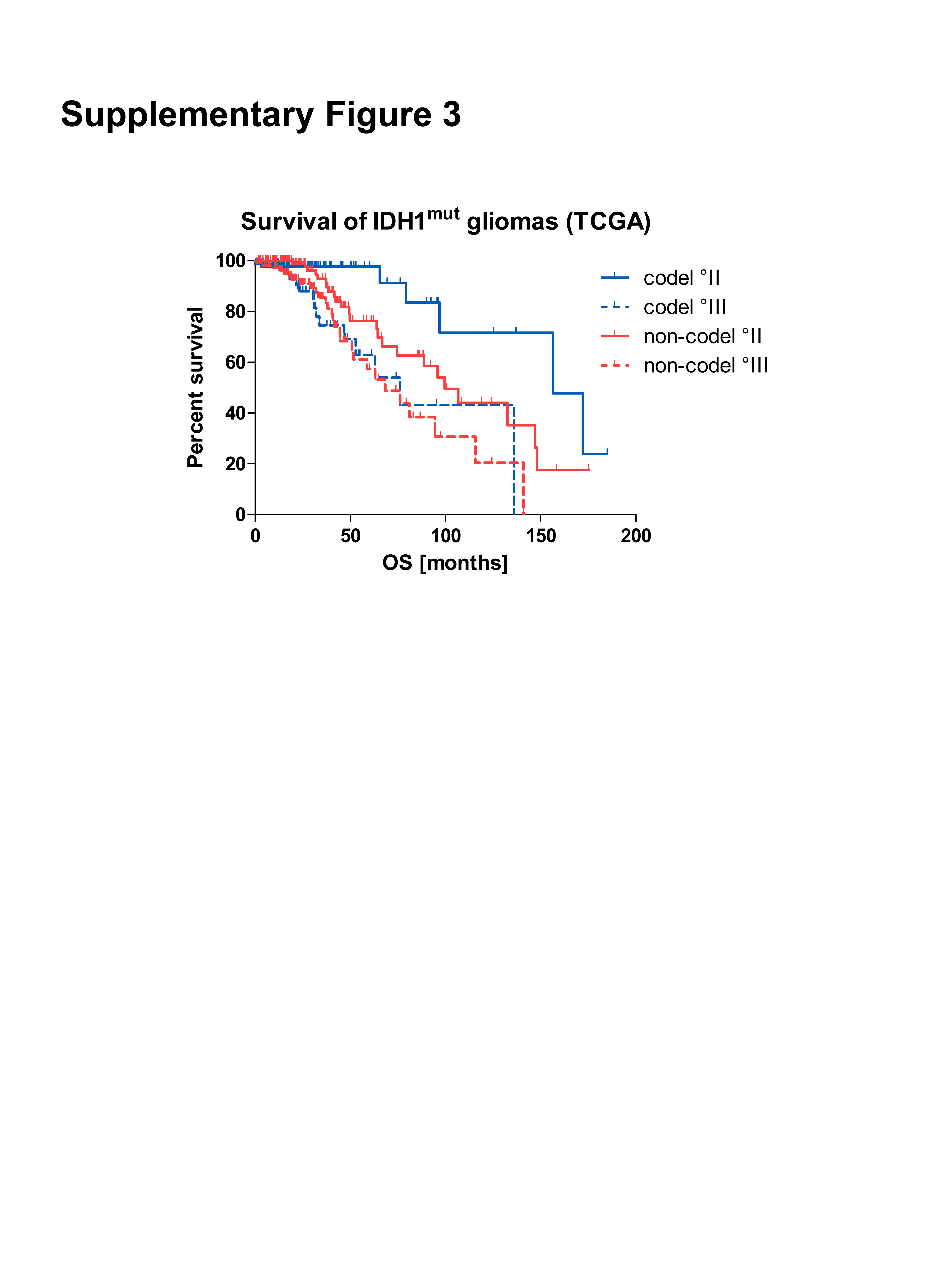

Supplement: Supplementary file 1 [file ijms-19-02903-s001.zip › SupplFigure3.jpg]
